# Structural and functional cardiac changes in myotonic dystrophy type 1: a cardiovascular magnetic resonance study

**DOI:** 10.1186/1532-429X-14-48

**Published:** 2012-07-24

**Authors:** Mieke CE Hermans, Catharina G Faber, Sebastiaan CAM Bekkers, Christine EM de Die-Smulders, Monique M Gerrits, Ingemar SJ Merkies, Gabriel Snoep, Yigal M Pinto, Simon Schalla

**Affiliations:** 1 Department of Neurology, Maastricht University Medical Centre, PO box 5800, Maastricht, AZ 6202, The Netherlands; 2 Department of Cardiology, Maastricht University Medical Centre, Maastricht, The Netherlands; 3 Department of Radiology, Maastricht University Medical Centre, Maastricht, The Netherlands; 4 Department of Clinical Genetics, Maastricht University Medical Centre, Maastricht, The Netherlands; 5 Department of Neurology, Spaarne Hospital, Hoofddorp, The Netherlands; 6 Department of Cardiology, Academic Medical Center, Amsterdam, The Netherlands

**Keywords:** Myotonic dystrophy, Cardiomyopathy, Cardiac magnetic resonance imaging, Endomyocardial fibrosis

## Abstract

**Background:**

Myotonic dystrophy type 1 (MD1) is a neuromuscular disorder with potential involvement of the heart and increased risk of sudden death. Considering the importance of cardiomyopathy as a predictor of prognosis, we aimed to systematically evaluate and describe structural and functional cardiac alterations in patients with MD1.

**Methods:**

Eighty MD1 patients underwent physical examination, electrocardiography (ECG), echocardiography and cardiovascular magnetic resonance (CMR). Blood samples were taken for determination of NT-proBNP plasma levels and CTG repeat length.

**Results:**

Functional and structural abnormalities were detected in 35 patients (44%). Left ventricular systolic dysfunction was found in 20 cases, left ventricular dilatation in 7 patients, and left ventricular hypertrophy in 6 patients. Myocardial fibrosis was seen in 10 patients (12.5%). In general, patients had low left ventricular mass indexes. Right ventricular involvement was uncommon and only seen together with left ventricular abnormalities. Functional or structural cardiac involvement was associated with age (p = 0.04), male gender (p < 0.001) and abnormal ECG (p < 0.001). Disease duration, CTG repeat length, severity of neuromuscular symptoms and NT-proBNP level did not predict the presence of myocardial abnormalities.

**Conclusions:**

CMR can be useful to detect early structural and functional myocardial abnormalities in patients with MD1. Myocardial involvement is strongly associated with conduction abnormalities, but a normal ECG does not exclude myocardial alterations. These findings lend support to the hypothesis that MD1 patients have a complex cardiac phenotype, including both myocardial and conduction system alteration.

## Background

Myotonic dystrophy type 1 (MD1), or Steinert’s disease, is an autosomal dominant inherited disorder caused by an unstable expansion of a repetitive trinucleotide sequence (CTG) on chromosome 19. The prevalence varies from 2.1-14.3 per 100 000 [1]. MD1 is characterized by slowly progressive weakness of skeletal muscles, myotonia and involvement of several organ systems [1]. An earlier age of onset and increased severity of clinical symptoms has been observed in subsequent generations and is related to degree of CTG expansion [2].

Patients with MD1 usually die from respiratory or cardiac complications [3,4]. Sudden death is considered to be the result of atrioventricular block or ventricular arrhythmias [5]. Recent studies showed that severe electrocardiographic (ECG) abnormalities and atrial arrhythmias are independent risk factors, although with moderate sensitivity, for sudden death in MD1 patients [6]. Although death from progressive heart failure is uncommon in patients with MD1 compared to other muscular dystrophies [7,8], left ventricular systolic dysfunction is associated with an increased risk of overall mortality and sudden death [9]. Therefore, the picture emerges that MD1 patients have a complex cardiac phenotype including both the myocardium and the conduction system.

Considering the importance of cardiomyopathy as a predictor of prognosis, we aimed to measure cardiac function and detect structural abnormalities in patients with MD1. We used cardiovascular magnetic resonance (CMR) in the current study since it is an accurate and highly reproducible technique for the assessment of cardiac volumes, function, mass and focal fibrosis and the interstudy reproducibility in normal, dilated, and hypertrophic hearts was superior to 2-dimensional echocardiography [10].

## Methods

### Patients selection

The protocol was approved by the local Medical Ethics Committee and each participant gave written informed consent. Patients older than 18 years of age were invited for a prospective, on-going study on cardiac involvement and early stratification of arrhythmogenic risk. Participants were recruited from the genetic register of the Maastricht University Medical Centre and through the Dutch neuromuscular patients’ association (Vereniging Spierziekten Nederland, VSN). Subjects with previously implanted pacemakers or implantable cardioverter-defibrillators or with severe comorbidity leading to reduced life expectancy such as malignant disease or respiratory failure, were excluded. During a 2 year period, 80 consecutive patients underwent CMR imaging and were enrolled in this study.

### Clinical examination

A standardized interview was conducted in all participants to evaluate their clinical history and current symptoms. A neurological and cardiac evaluation was conducted by the same examiner (MH) in a predefined standardized fashion. The MD1 phenotype was established according to the commonly accepted classification based on the age at onset of symptoms: mild (late onset), classical (adult onset) and childhood/congenital type [2]. Skeletal muscle strength was manually tested and graded according to the Medical Research Council (MRC) 6-point grading system (0–5) [11]. A total of 22 muscle groups were tested: neck flexors and extensors separately plus 10 bilateral muscles: 6 proximal muscle groups (shoulder abductors, elbow flexors, elbow extensors, hip flexors, knee extensors, knee flexors) and 4 distal muscle groups (wrist extensors, digits flexors, ankle dorsiflexors, ankle plantar flexors). Summation of the scores yields an extended MRC-sumscore, ranging from 0 (paralytic) to 110 (normal strength) [12,13]. Furthermore, blood samples were taken to determine N-Terminal pro Brain Natriuretic Peptide (NT-pro-BNP) plasma levels and the length of the CTG repeat.

### CTG repeat length analysis

Analysis of the MD1 CTG repeat length was performed on peripheral blood lymphocytes. Polymerase chain reaction followed by fragment length analysis was used to determine small allele lengths of 5 to 100 repeats, and Southern blotting was used to estimate repeat lengths >100. For purposes of statistical analysis, the CTG expansions were divided in 4 categories (<100; 100–250; 250–500; >500).

### Electrocardiography

ECG was considered abnormal if signs of conduction disease (PR interval ≥210 ms, QRS duration ≥120 ms, left anterior or posterior fascicular hemiblock), hypertrophy (Sokolow-Lyon index ≥35 mm), myocardial infarction or rhythm other than sinus, were present.

### Echocardiography

Echocardiography was used to exclude significant valvular disease, elevated right ventricular systolic pressure. Transthoracic echocardiograms were performed using a SONOS 5500 system with S3 transducer (Philips Medical Systems, Best, The Netherlands). Echocardiographic investigations were performed according to the recommendations of the American Society of Echocardiography.

### CMR

Patients were examined in supine position with a clinical 1.5 T Gyroscan Intera MR scanner (Philips Medical Systems, Best, The Netherlands) equipped with a 5 channel cardiac surface coil. ECG-gated cine images were acquired for functional analysis during multiple breath holds (10–13 seconds) using a steady-state free precession sequence (slice thickness 6 mm, slice gap 4 mm, TR/TE 3.8/1.9 ms, flip angle 50^°^, FOV 350 mm, matrix 256 x 256, 22–25 phases per cardiac cycle) in two-chamber, three-chamber and four-chamber view and a short-axis stacks covering the entire LV. For the detection of myocardial edema multislice short axis images were acquired using a dual-inversion black-blood T2-weighted sequence with fat suppression (slice thickness 8 mm, slice gap 2 mm, TR/TE 1600/100 ms, flip angle 90^°^, FOV 350 mm, matrix 512 x 512). After intravenous contrast administration (Gd-DTPA 0.2 mmol/kg) a Look-Locker sequence (slice thickness 10 mm, TR/TE 3.6/1.7 ms, flip angle 8^°^, FOV 370 mm, resolution 256 x 256, 39 phases, phase interval 15 ms) was applied to determine the inversion time (TI) to optimally “null” LV myocardium (typical TI range 200–280 ms) for the subsequent scan. To evaluate the presence of myocardial late gadolinium enhancement (LGE) a breath-hold 3D inversion recovery gradient echo sequence covering the entire LV (acquired slice thickness 12 mm, reconstructed slice thickness 6 mm, average TR/TE 3.9/2.4 ms, multishot (50 profile/shot) segmented partial echo readout every heartbeat, flip angle 15, field of view 400 mm, matrix 256 x 256, acquired and reconstructed pixel size 1.56 x 1.56 mm, typically 16–18 slices) was used with images in short-axis, two-chamber and four-chamber view, acquired 10 minutes after the administration of intravenous contrast.

### CMR data analysis

MR images were analyzed with commercially available software (CAAS MRV 3.0, Pie medical imaging, Maastricht, The Netherlands). Endocardial and epicardial contours were manually traced in end-diastolic and end-systolic phases on short axis cine images to determine end-diastolic and end-systolic volume, ejection fraction and LV end-diastolic mass. Systolic LV dysfunction was defined as an ejection fraction <55% or regional wall motion abnormalities. LV and right ventricular (RV) dilatation were defined as enddiastolic volumes >2SD and RV systolic dysfunction as ejection fraction <2SD of mean reference values normalized for gender, body surface area and age [14]. We considered LV hypertrophy as increase in the LV mass and LV wall hypertrophy as wall thickness >12 mm. The presence and localization of edema or focal fibrosis was visually identified by a consensus of two independent experienced observers using the T2-weighted and LGE images. CMR was considered to be abnormal if regional or global dysfunction, ventricular dilatation, hypertrophy, or areas of fibrosis or edema were observed.

### Statistical analysis

Descriptive statistics of clinical characteristics, electrocardiographic findings and cardiac magnetic resonance results are presented. Categorical variables were summarized by frequency counts (percentage) and differences between groups were evaluated using chi-square test. For continuous variables, results are presented as median (range) and comparison between categories was made with Mann–Whitney *U* test. Multiple group comparisons were made with Kruskal-Wallis test. All analyses were performed using SPSS software version 15.0. A probability (p) value of <0.05 at a two-sided level was considered statistically significant.

## Results

### Patient characteristics

Characteristics of 80 MD1 patients (all Caucasian; 45 males and 35 females) are shown in Table 1. No significant differences in clinical characteristics were found between men and women. As expected, with increasing CTG repeat length, the median age at onset and skeletal muscle strength score decreased (p < 0.001). All patients were ambulant for short distances (<100 meter), but 16 subjects used mobility aids and 4 patients were confined to a wheelchair for longer distances. Fatigue and dyspnoea were frequently reported symptoms: 48 patients were either dyspnoeic or fatigued after exertion and 10 subjects complained of both. No patient reported a history of syncope, severe palpitations at rest, angina pectoris or myocardial infarction. Mild peripheral edema was seen in four patients, all of whom had normal systolic and diastolic LV function and normal NT-proBNP levels.

**Table 1:** Clinical and genetic characteristics according to clinical phenotype category

	**Mild type n = 9**	**Classical type n = 63**	**Congenital/childhood type n = 8**	**Total n = 80**
Male	5 (56%)	33 (52%)	7 (88%)	45 (56%)
Age in years (range)	60 (46–70)	47 (24–64)	32 (24–51)	48 (24–70)
Age at onset in years (range)	52 (50–65)*	27 (10–51)	6.5 (0–10)	27 (0–65)
Muscle strength sumscore (range)	110 (107–110)	96 (73–109)	100 (78–109)	98 (73–110)
Abnormal ECG	1 (11%)	41 (65%)	7 (88%)	49 (61%)
Abnormal CMR	3 (33%)	28 (44%)	4 (50%)	35 (44%)

*age at onset of neuromuscular signs and symptoms could not be determined in 3 patients, since they were still asymptomatic.

### CMR

**Figure 1:**
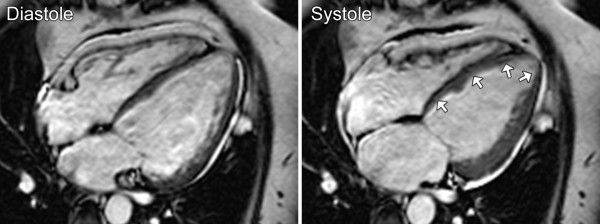
**Ventricular dysfunction in myotonic dystrophy type 1 by CMR.** Cine images in four-chamber long-axis view in diastole and systole of a patient with impaired systolic left ventricular function (ejection fraction 38%): septal and apical hypokinesia (arrows).

Functional or structural abnormalities were detected with CMR in 35 patients (44%). The results of CMR analysis are summarized in Table 2. LV systolic dysfunction was found most frequently, being present in 20 patients. An example of LV dysfunction on CMR is shown in Figure 1. Concomitant dilatation of the LV was found in 4 patients, while 3 patients had LV dilatation with preserved systolic function. Regional LV hypokinesia was observed in 11 patients and co-localized with local thinning of the wall in 3 cases. LV hypertrophy was observed in 6 patients. None of the patients with LV hypertrophy had arterial hypertension. LV mass indexes of MD1 patients were remarkably low and the mean values differed significantly from values obtained from healthy volunteers (*t*-test, p < 0.001) [14]. Right ventricular dysfunction or dilatation was only present in patients with LV dysfunction. Abnormal myocardial function and structure was more frequent in men than in women (p < 0.001) and associated with higher age (p = 0.04), but not with duration of disease, muscle strength sumscore or CTG repeat length.

**Table 2:** CMR results

	**All n = 80**	**Male n = 45**	**Female n = 35**
LV ejection fraction, % (range)	58 (38–73)	57 (45–73)	61 (38–71)
LV systolic dysfunction, n (%)	20 (25%)	16 (36%)	4 (11%)
LV enddiastolic volume, ml/m^2^ (range)	72 (38–117)	77 (41–117)	67 (38–104)
LV endsystolic volume, ml/m^2^ (range)	31 (11–63)	35 (14–63)	28 (11–56)
LV dilatation, n (%)	7 (9%)	6 (13%)	1 (3%)
LV mass, g/m^2^ (range)	47(30–79)	50 (36–79)	41(30–67)
LV wall hypertrophy, n (%)	6 (8%)	4 (9%)	2 (6%)
RV ejection fraction, % (range)	64 (38–77)	60 (38–77)	67 (50–76)
RV systolic dysfunction, n (%)	4 (5%)	4 (9%)	0
RV enddiastolic volume, ml/m^2^ (range)	66 (40–117)	71 (40–117)	61 (40–102)
RV endsystolic volume, ml/m^2^ (range)	23 (10–66)	28 (10–66)	20 (10–46)
RV dilatation, n (%)	1 (1%)	1 (2%)	0
RV outflow tract, mm (range)	26 (21–37)	27 (22–37)	25 (21–29)
Myocardial fibrosis, n (%)	10 (13%)	7 (16%)	3 (9%)

**Figure 2:**
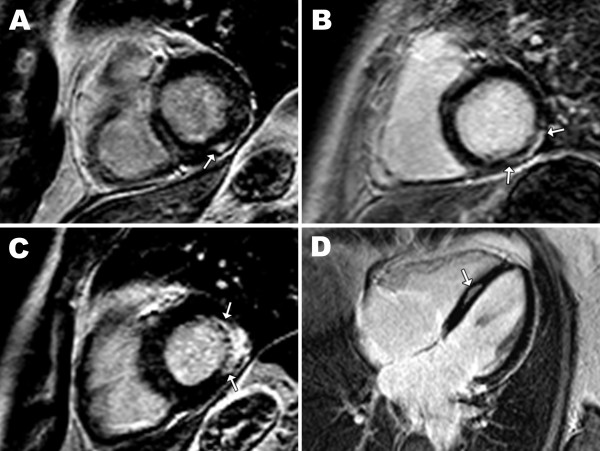
**Myocardial fibrosis in myotonic dystrophy type 1 by CMR.** Late gadolinium enhancement (LGE) images in short axis (**A**, **B** and **C**) and 4-chamber long axis views (**D**) of 4 patients with myotonic dystrophy type 1. Between arrows are regions of increased signal intensity, indicating focal fibrosis, visible as mid-myocardial enhancement to epicardial enhancement with endocardial sparing.

Focal myocardial fibrosis was detected on LGE images in 10 patients, most often as midmyocardial enhancement of the septal segments and basal (inferio) lateral segments of the LV wall (n = 8). Subendocardial and partly transmural enhancement of the basal lateral wall was also found (n = 2). Examples of selected CMR images are shown in Figure 2. No patient had signs of myocardial edema on T2-weighted images. No significant relationship between the presence of myocardial fibrosis and MD1 phenotype or CTG repeat length was observed.

### ECG and echocardiography

Electrocardiographic findings are summarized in Table 3. An abnormal ECG was recorded in 49 patients (61%). All patients were in sinus rhythm except for 2 with atrial fibrillation. Conduction delay was present in 46 patients (58%) and 1 patient had abnormal Q-waves. There were no signs of hypertrophy on the ECGs. In general, patients with rhythm or conduction disturbances had more severe skeletal muscle weakness than those without (p = 0.002). Of the 31 patients with normal ECGs, seven showed sinusbradycardia without conduction abnormalities (frequency 50–60 bpm, n = 4; frequency <50 bpm, n = 3).

**Table 3:** Electrocardiography results

	**n = 80**
Frequency, bpm (range)	70 (40–95)
Sinusbradycardia, n (%)	14 (18%)
Atrial fibrillation, n (%)	2 (3%)
PR interval, ms (range)	200 (136–460)
Prolonged PR interval, n (%)	30 (38%)
QRS duration, ms (range)	100 (80–164)
Intraventricular conduction delay, n (%)	26 (33%)
Left anterior fascicular block, n (%)	6 (8%)
Left posterior fascicular block, n (%)	1 (1%)

* sinusbradycardia was defined as frequency <60 bpm.

Echocardiography ruled out hemodynamically significant valvular disease or elevated right ventricular pressures in all patients.

**Figure 3:**
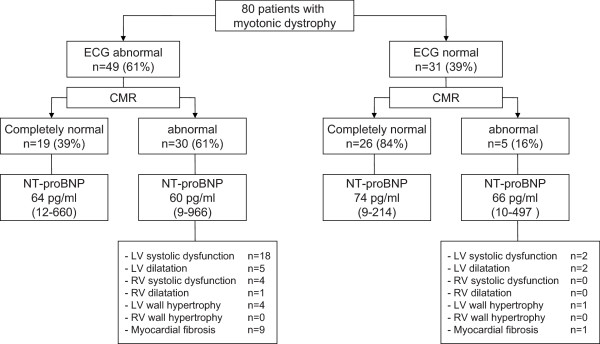
**Graphic reproduction of cardiac evaluation in myotonic dystrophy patients showing ECG and CMR findings.** The majority of patients with ECG abnormalities had functional or structural cardiac abnormalities. However, a substantial number of patients with normal ECG also showed myocardial alterations. NT-proBNP levels did not help to distinguish between patients with and without impaired myocardial functioning (presented as median (range)).

A graphic reproduction of the cardiac evaluation is shown in Figure 3. There was an association between ECG abnormalities and abnormal CMR findings (p < 0.001). Patients with an abnormal ECG were more likely to have functional or structural cardiac abnormalities (Odds ratio 8.2; 95% CI 2.7-25.1). However, myocardial involvement was also seen in 5 out of 31 patients with a normal ECG. The sensitivity of the ECG to predict myocardial involvement in this selected population was 86%, with a specificity of 58%. Late gadolinium enhancement was found in 9 out of 49 patients with ECG abnormalities and only 1 of the 31 patients with a normal ECG. NT-proBNP levels did not significantly differ between patients with and without cardiac conduction disease or myocardial abnormalities. Complaints of fatigue and dyspnoea on exertion were not associated with abnormalities on ECG or imaging.

## Discussion

The principal finding of this study is that structural and functional myocardial abnormalities are frequent in MD1 patients. The presence of mild to moderate left ventricular systolic dysfunction, ventricular dilatation, myocardial hypertrophy or fibrosis was strongly associated with electrocardiographic conduction abnormalities. However, 16% of patients with a normal ECG still had myocardial alterations. These findings lend support to the concept that the myocardium is generally involved in the pathogenic process of MD1.

Myocardial involvement may be prognostic in predicting death in MD1 [9]. CMR is well established in cardiomyopathies, because of its greater sensitivity and reproducibility than conventional diagnostic investigations (ECG and echocardiography) to demonstrate early abnormalities or subtle changes [15]. However, the CMR phenotype of MD1 had not been well characterized. Initial descriptions of CMR findings in MD1 revealed structural abnormalities, but the number of investigated subjects was small and functional analysis had not been carried out [16]. Increased left ventricular trabeculation confirmed by CMR has been reported in two related patients with MD1 [17]. Another study described a possible relationship between CMR abnormalities of the right ventricle and inducible arrhythmias at electrophysiological testing in MD1 patients [18]. Yet, no gadolinium contrast was used to visualize fibrosis and the induced ventricular arrhythmias were mostly non-sustained. We did not find any isolated remarkable abnormalities of the right ventricle or left ventricular trabeculation in our large cohort.

Fibrosis is a frequent histopathological finding in individual MD1 cases [19-23]. Focal myocardial fibrosis as detected by LGE-CMR was present in 13% of our patients. As in other non-ischemic cardiomyopathies, late gadolinium enhancement was usually located in the interventricular septum and often limited to the mid-myocardium [24,25]. An increased risk of sustained ventricular tachycardia and sudden death is associated with midwall fibrosis in patients with dilated cardiomyopathy [26]. Whether midwall fibrosis determined by CMR is a predictor of mortality in MD1 remains to be investigated.

The low prevalence of symptomatic heart failure in MD1 is usually partly attributed to the reduced cardiac demand due to diminished skeletal muscle activity [27]. This lower hemodynamic load in MD1 patients can also explain the low LV mass indexes found in this study.

Increasing age, male sex and ECG conduction abnormalities are all significantly associated with myocardial disease, whereas CTG repeat length and severity of muscular impairment are not. Male gender and age have been positively associated with arrhythmia and conduction abnormalities [28]. There is no consensus from the literature as to whether or not CTG repeat size has value as a prognostic indicator of conduction disturbances or cardiac events [28-30]. While age at onset of symptoms and severity of the phenotype correlate with the size of the CTG repeat, the association between the length of the CTG repeat measured in leukocytes and other symptoms of MD1 is more elusive. The heterogeneity of symptoms shown by patients with similar CTG repeat sizes can partly be explained by the presence of somatic mosaicism and somatic expansion over time [31].

Structural and functional cardiac changes were found in patients with mild as well as severe neurological phenotypes. Duration of neuromuscular disease was not significantly related to cardiac disease, indicating that cardiac manifestations can precede, coincide with or succeed skeletal myopathy. It should however be stressed that recall of age at onset is sometimes poor and unreliable, as the diagnosis is often considerably delayed. Furthermore, duration of symptoms do not necessarily relate to the severity of neuromuscular symptoms as disease progression is highly variable. Symptoms of dyspnoea or fatigue were not associated with LV dysfunction and may therefore largely be ascribed to the progressive physical disability of the muscular disease.

The current study is a descriptive study of a large cohort of patients with MD1 using state of the art diagnostic technology. A limitation of the descriptive survey is the absence of a comparison group or prognostic data allowing no inferences to be drawn about cause of disease and the predictive value of myocardial fibrosis or other CMR findings for identifying patients with MD1 who are at risk for cardiac death.

## Conclusions

Subclinical cardiomyopathy in patients with MD1 is frequently observed with CMR. Screening for functional and structural cardiac disease should be considered in all patients since myocardial involvement can be overlooked by ECG alone. Whether the identification of structural or functional cardiac changes has prognostic implications for the prediction of disease progression or sudden death remains to be investigated in a long-term prospective study.

## Abbreviations

CI, Confidence interval; CMR, Cardiovascular magnetic resonance; CTG, Cytosine-thymine-guanine; MD1, Myotonic dystrophy type 1; ECG, Electrocardiogram; LGE, Late gadolinium enhancement; MRC, Medical research council; NT-proBNP, N-terminal prohormone of brain natriuretic peptide; LV, Left ventricle; OR, Odds ratio; RV, Right ventricle; SD, Standard deviation.

## Competing interests

The authors declare that they have no competing interests.

## Authors’ contribution

All authors have contributed significantly to the work and have read and approved the manuscript. CF, CD-S and YP designed the study. Cardiac magnetic resonance imaging studies were analyzed by SS, SB and GS. MG did the analysis of the DMPK gene CTG repeat. Collection and interpretation of other data was done by IM and MH. Eventually, MH drafted the manuscript and all authors made critical revisions.
